# Cardiac arrhythmia and hypoglycaemia among individuals with and without diabetes receiving haemodialysis (the CADDY study): a Danish multicentre cohort study

**DOI:** 10.1007/s00125-025-06388-5

**Published:** 2025-02-28

**Authors:** Dea H. Kofod, Søren Z. Diederichsen, Tobias Bomholt, Mads Ø. Andersen, Andreas Andersen, Ebba Mannheimer, Marianne Rix, Ylian S. Liem, Kristine Lindhard, Henrik P. Hansen, Casper Rydahl, Morten Lindhardt, Julie Brøsen, Kristine Schandorff, Theis Lange, Kirsten Nørgaard, Thomas P. Almdal, Jesper H. Svendsen, Bo Feldt-Rasmussen, Mads Hornum

**Affiliations:** 1 Department of Nephrology and Endocrinology, Copenhagen University Hospital – Rigshospitalet, Copenhagen, Denmark; 2 Department of Cardiology, Copenhagen University Hospital – Rigshospitalet, Copenhagen, Denmark; 3 Clinical Research, Copenhagen University Hospital – Steno Diabetes Center Copenhagen, Herlev, Denmark; 4 Department of Nephrology, Copenhagen University Hospital – Herlev and Gentofte, Herlev, Denmark; 5 Department of Internal Medicine, Copenhagen University Hospital – Holbeak, Holbeak, Denmark; 6 Department of Clinical Medicine, Faculty of Health and Medical Sciences, University of Copenhagen, Copenhagen, Denmark; 7 Department of Endocrinology and Nephrology, Copenhagen University Hospital – North Zealand, Hilleroed, Denmark; 8 Section of Biostatistics, University of Copenhagen, Copenhagen, Denmark

**Keywords:** Arrhythmia, End-stage kidney disease, Haemodialysis, Hypoglycaemia, Type 1 diabetes, Type 2 diabetes

## Abstract

**Aims/hypothesis:**

We aimed to examine arrhythmias and hypoglycaemia among individuals with and without diabetes who are receiving haemodialysis and to investigate the association between arrhythmias and hypoglycaemia, hyperglycaemia and glycaemic variability.

**Methods:**

This prospective multicentre cohort study included 70 participants on maintenance haemodialysis (35 with diabetes and 35 without diabetes). We employed implantable cardiac monitors for continuous heart rhythm monitoring in combination with periodic use of continuous glucose monitoring. Logistic-regression-type linear mixed models were used to examine associations between arrhythmias and glycaemic measures.

**Results:**

During 18 months of follow-up, clinically significant arrhythmias (bradyarrhythmia and ventricular tachycardia) were identified in 12 (34%) participants with diabetes and 11 (31%) without diabetes. Atrial fibrillation was detected in 13 (37%) participants with diabetes and 14 (40%) without, while other supraventricular tachycardia was detected in seven (20%) and 11 (31%) participants with and without diabetes, respectively. Hypoglycaemia (sensor glucose <3.9 mmol/l) was observed in 27 (77%) participants with diabetes and 32 (91%) without diabetes. Compared with euglycaemia, hypoglycaemia was associated with an increased rate of arrhythmias among participants without diabetes (incidence rate ratio [IRR] 3.13 [95% CI 1.49, 6.55]), while hyperglycaemia (sensor glucose >10.0 mmol/l) was associated with a decreased rate of arrhythmias among participants with diabetes (IRR 0.58 [95% CI 0.37, 0.92]). Glycaemic variability showed no association with arrhythmias regardless of the presence of diabetes.

**Conclusions/interpretation:**

Arrhythmias and hypoglycaemia were common in those undergoing haemodialysis regardless of diabetes status. Our data suggest a temporal relationship between arrhythmias and glucose level in both individuals with and without diabetes.

**Trial registration:**

Clinicaltrials.gov: NCT04841304.

**Graphical Abstract:**

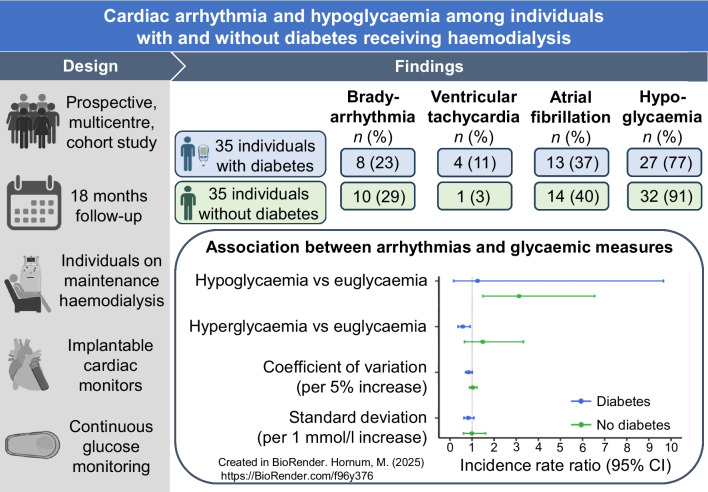

**Supplementary Information:**

The online version of this article (10.1007/s00125-025-06388-5) contains peer-reviewed but unedited supplementary material.


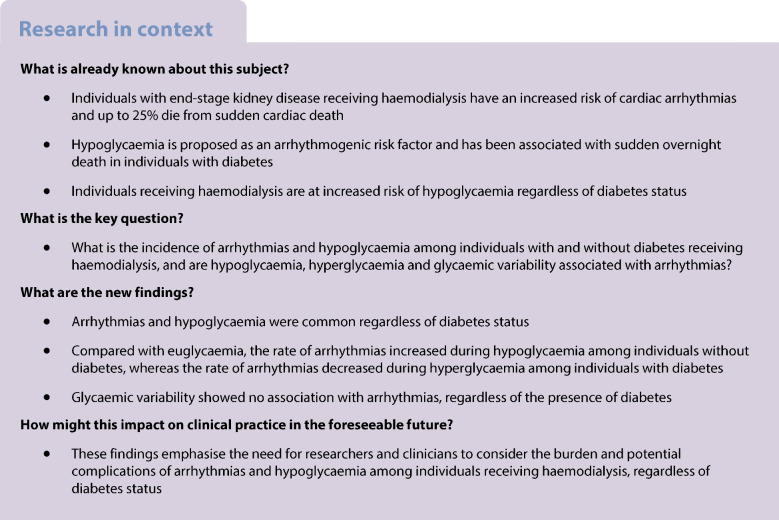


## Introduction

Individuals with end-stage kidney disease receiving haemodialysis are at increased risk of cardiac arrhythmias [1, 2]. The increased arrhythmogenicity may contribute to the high incidence of cardiovascular mortality observed in this population, particularly sudden cardiac death, which accounts for up to 25% of all deaths among individuals receiving haemodialysis [3, 4]. However, data on the incidence of specific arrhythmias and their underlying mechanisms remain limited [1, 2].

Hypoglycaemia is proposed as a trigger of lethal arrhythmias and has been associated with sudden overnight death in individuals with diabetes [5]. Several clinical studies have linked hypoglycaemia to ECG changes and arrhythmias. Among individuals with and without diabetes who have preserved kidney function, experimentally induced hypoglycaemia has been associated with abnormal cardiac repolarisation, which may increase the risk of ventricular arrhythmias [6–8]. Furthermore, an association has been found between nocturnal hypoglycaemia and bradycardia in two studies employing 4–5 days of simultaneous Holter monitoring and continuous glucose monitoring (CGM) in individuals with type 1 and type 2 diabetes [9, 10]. Another study employing 5 days of simultaneous Holter monitoring and CGM in individuals with type 2 diabetes reported an increased risk of ventricular tachycardia among those with hypoglycaemic events [11].

Two recent studies performed long-term monitoring of heart rhythm using implantable loop recorders (ILRs) in combination with CGM [12, 13]. These studies included 30 individuals with type 1 diabetes (13% with chronic kidney disease [CKD]) and 21 individuals with insulin-treated type 2 diabetes (43% with CKD), respectively. They found no clear association between hypoglycaemia and arrhythmias, although arrhythmias were associated with daytime hyperglycaemia in those with type 1 diabetes and with glycaemic variability in those with type 2 diabetes.

It has been proposed that the counterregulatory responses activated during hypoglycaemia to restore normal glucose levels are also responsible for triggering arrhythmias through the impact of the autonomic nervous system on the heart [5]. Individuals on haemodialysis may be particularly vulnerable to hypoglycaemia because of a high prevalence of underlying predisposing factors to arrhythmias, such as functional and structural heart disease, autonomic dysregulation and factors related to kidney failure and the dialysis procedure itself, including fluid, acid–base and electrolyte excursions [2, 14–16]. Moreover, these individuals are at increased risk of hypoglycaemia regardless of diabetes status [17, 18]. Thus, hypoglycaemia may be an important risk factor for arrhythmias in those undergoing haemodialysis.

To our knowledge, no previous studies have examined the association between arrhythmias and hypoglycaemia or other glycaemic measures among individuals with and without diabetes receiving haemodialysis. This study employed long-term follow-up with ILRs and CGM simultaneously to examine the incidence of cardiac arrhythmias and hypoglycaemia among individuals with and without diabetes who are receiving haemodialysis and to investigate the association between arrhythmias and hypoglycaemia, hyperglycaemia and glycaemic variability.

## Methods

### Study design and population

Cardiac arrhythmia and hypoglycaemia among individuals with and without diabetes receiving haemodialysis (the CADDY study) was an investigator-initiated, prospective, observational, multicentre, cohort study that investigated individuals with and without diabetes who received haemodialysis from four Danish dialysis centres (Rigshospitalet, Herlev Hospital, North Zealand Hospital and Holbeak Hospital). Participants were monitored with ILRs for 18 months of follow-up, and CGM was applied for 10 days at baseline and every 2 months during follow-up. The study protocol has been published previously [19].

We included individuals ≥18 years of age receiving chronic hospital-based outpatient maintenance haemodialysis for ≥3 months. Participants with diabetes included both those who had type 1 diabetes and those who had type 2 diabetes who were on treatment with glucose-lowering medication at inclusion. Participants without a diabetes diagnosis had no record of previous glucose-lowering treatment and an HbA_1c_ level <48 mmol/mol (6.5%) at screening. The following exclusion criteria were applied: (1) cardiac pacemakers or implantable cardioverter-defibrillators; (2) known permanent (chronic) atrial fibrillation; (3) history of sustained (>30 s) ventricular tachycardia (more than 200 beats/min) or ventricular fibrillation; (4) known cardiac ion-channel disease; (5) previous complications with wearing a CGM sensor; or (6) contraindications for ILR implantation. Sex was not considered in the study design, and both men and women were included in the study. All participants were scheduled for a baseline visit where data were obtained based on information from the participants and their medical records. Sex and ethnicity were self-reported.

### Cardiac rhythm monitoring

All participants were monitored with an ILR (Reveal LINQ; Medtronic, Minneapolis, USA), which is a small wireless cardiac monitor. The ILR was implanted on the left side of the chest under local anaesthesia at the baseline visit. When implanted, the ILR continuously recorded a one-lead ECG to detect arrhythmias according to the following prespecified algorithm: bradycardia (≥4 beats at rate <30 beats/min); pause (>3 s); tachycardia (≥16 beats at rate >150 beats/min); and atrial fibrillation (≥2 min). The recorded ECGs were automatically transmitted and then manually reviewed by the study personnel.

### CGM

Using the Dexcom G6 system (Dexcom, San Diego, USA), CGM data were collected from all participants for 10 days at baseline and every 2 months during follow-up (nine CGM periods per participant). CGM measured interstitial glucose level every 5 min; the sensor was placed on the back of the upper arm by study personnel during a regularly scheduled dialysis session. CGM data were blinded and the study team did not access the data during follow-up. Participants with diabetes who were already using a CGM system (other than the Dexcom G6 system) before the study inclusion continued with their usual device, and the blinded study CGM was still applied at baseline and every 2 months. However, participants who were already using the Dexcom G6 system before the study inclusion were invited to share their data with the study team, and 10 days of data were downloaded at baseline and every 2 months without applying the blinded study CGM.

### Study outcomes and follow-up

The primary outcome was the incidence of clinically significant arrhythmias, defined as a composite outcome of significant bradycardia (pause >3 s or ≥4 beats with rate <30 beats/min), ventricular tachycardia (sustained [rate >150 beats/min lasting ≥30 s] or non-sustained [rate >150 beats/min lasting <30 s]) or ventricular fibrillation. Secondary outcomes included the incidence of atrial fibrillation (lasting ≥2 min) and other supraventricular tachycardia (rate >150 beats/min lasting ≥16 beats). ‘Any arrhythmia’ was defined as any of the five defined subtypes of arrhythmias.

Glycaemic characteristics derived from CGM data were reported based on international consensus guidelines [20]. Hypoglycaemic events were defined as sensor glucose <3.9 mmol/l for ≥15 consecutive minutes, and an event ended when there were ≥15 consecutive minutes with a CGM sensor value of ≥3.9 mmol/l [20]. Level 2 hypoglycaemic events were defined as sensor glucose <3.0 mmol/l for ≥15 consecutive minutes. Hyperglycaemic events were defined as sensor glucose >10.0 mmol/l for ≥15 consecutive minutes, and an event ended when there were ≥15 consecutive minutes with a CGM sensor value of ≤10.0 mmol/l. Euglycaemia was defined as sensor glucose of 3.9–10.0 mmol/l. Glycaemic variability was reported as the SD of the mean glucose and the percentage CV, which was calculated as 100 × (SD divided by mean glucose). The participants were followed for 18 months or until the time of withdrawal, kidney transplantation, dialysis modality change or death, whichever occurred first.

### Statistical analyses

Given a previously reported incidence of 60% for significant arrhythmias in individuals undergoing haemodialysis, a sample size of 70 individuals was selected to estimate the incidence of clinically significant arrhythmias with a precision of ±11 percentage points (i.e. the width of the 95% CI). Data were reported as counts with percentages for categorical data and as mean with SD or median with IQR for continuous data. Differences in baseline data were compared between participants with and without diabetes using Fisher’s exact test for categorical data and a two-sample* t* test or Wilcoxon test for continuous data.

The association between arrhythmias (any arrhythmia) and glycaemic measures (hypoglycaemia, hyperglycaemia and glycaemic variability) was analysed for the entire sample and for the groups with and without diabetes separately. Using the same approach as two similar studies [12, 13], the aim was to examine the association between arrhythmias and each glycaemic measure in continuous time with an estimation of the effect of the within-subject changes in the glycaemic measure separate from the effect of the between-subject variation in glycaemic characteristics, which is prone to confounding. To this end, the occurrence of arrhythmic events (based on the onset) was summarised per hour and analysed as a binary outcome (hours with event[s] vs no event). Similarly, each of the following glycaemic measures was summarised per hour: occurrence of hypoglycaemic events (binary: hours with event vs no event); occurrence of hyperglycaemic events (binary: hours with event vs no event); time spent in the hypoglycaemic event (per 5 min); time spent in the hyperglycaemic event (per 5 min); and Δ plasma glucose (the difference between the peak and nadir values over a 2 h period, including the preceding and current hour).

To separate the between-subject effect from the within-subject effect, the mean of each glycaemic measure over the study period and the hourly deviations from the mean of each measure were computed for each participant [21]. These variables were then used as fixed effects in a logistic-regression-type generalised linear mixed model, which included participant ID as a random effect to account for repeated measures within participants. Based on this, we calculated subject-specific ORs for within-subject effects with 95% CI. Subject-specific ORs were interpreted as incidence rate ratios (IRRs) due to the low occurrence of arrhythmic events during simultaneous CGM.

The effect of hypoglycaemia was compared with euglycaemia and hyperglycaemia combined and with euglycaemia alone (excluding hours with hyperglycaemic events). In the analyses of glycaemic variability, we used the same approach as described above but summarised arrhythmic events, CV and SD per day (00:00–24:00 hours) and also included night-time (00:00–06:00 hours) as a fixed effect in the model. In addition, all analyses were repeated with stratification by daytime (06:00–24:00 hours) and night-time (00:00–06:00 hours). Due to the exploratory nature of the study, *p* values were not reported.

### Additional analyses

To examine a potential association between arrhythmias (any arrhythmia) and baseline use of beta-blockers or selective calcium blockers, we used a negative binomial regression model (due to overdispersion) with the arrhythmic count during follow-up as the outcome, adjusted for a history of paroxysmal atrial fibrillation and differences in follow-up time.

### Ethics

The study was approved by the Scientific Ethics Committee of the Capital Region of Denmark (H-20069767) and the Knowledge Centre on Data Protection of the Capital Region of Denmark. It is registered at ClinicalTrials.gov (trial registration no. NCT04841304). All participants provided oral and written informed consent prior to inclusion.

## Results

**Fig. 1:**
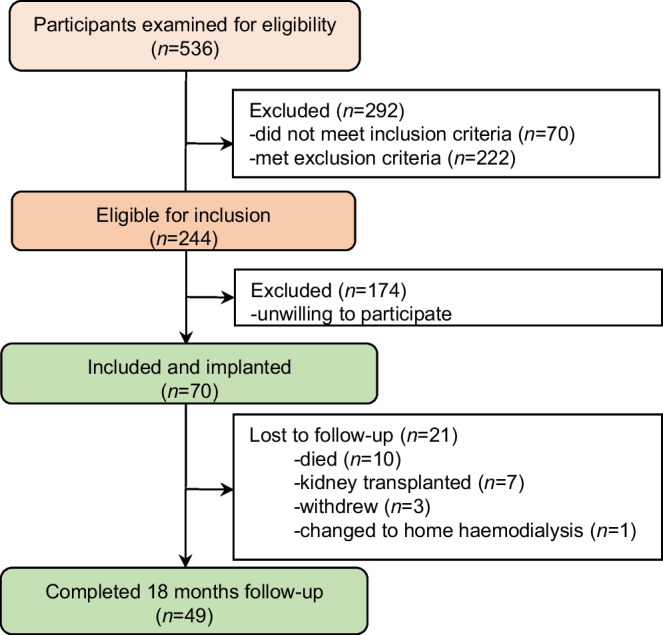
Flow chart of inclusion and follow-up

From June 2021 to October 2022, 536 individuals on haemodialysis were screened for inclusion and 70 of them were enrolled, including 35 with diabetes and 35 without diabetes (Fig. 1). The median follow-up time was 18 months (IQR 15–18 months). The total follow-up time was similar for those with diabetes (538 follow-up months) and those without diabetes (535 follow-up months). There were 49 participants who completed 18 months of follow-up. The most frequent reasons for not completing follow-up were death (*n*=10) and kidney transplantation (*n*=7). All data obtained before the participants exited the study were included in the analyses.

### Baseline characteristics

The baseline characteristics of the participants are presented in Table 1. The median age was 67 years (IQR 58–75 years), 27 (39%) were women and 19 (27%) had a history of paroxysmal atrial fibrillation. None of the participants had a history of clinically significant arrhythmia or supraventricular tachycardia other than atrial fibrillation. Table 2 shows the diabetes-related baseline characteristics of the participants with diabetes. There were seven (20%) participants who had type 1 diabetes and 30 (86%) who received insulin treatment. No participants were treated with sulfonylureas. Baseline pre-dialysis blood sample measures and dialysis parameters are presented in Table 3.

**Table 1:** Baseline characteristics

Characteristic	All (*n*=70)	Diabetes (*n*=35)	No diabetes (*n*=35)	*p* value
Age, years	67 (58–75)	66 (58–74)	69 (55–75)	0.92
Sex				0.05
Women	27 (39)	18 (51)	9 (26)	
Men	43 (61)	17 (49)	26 (74)	
Ethnicity				0.61
White	65 (93)	32 (91)	33 (94)	
Arab	2 (2.9)	2 (5.7)	0 (0)	
Asian	3 (4.3)	1 (2.9)	2 (5.7)	
Smoking status				0.66
Never	26 (37)	15 (43)	11 (31)	
Past	33 (47)	15 (43)	18 (51)	
Current	11 (16)	5 (14)	6 (17)	
Cause of end-stage kidney disease				<0.01
Diabetes	27 (39)	27 (77)	0 (0)	
Hypertension	7 (10)	0 (0)	7 (20)	
Glomerulonephritis	7 (10)	3 (8.6)	4 (11)	
Other/unknown	29 (41)	5 (14)	24 (69)	
Duration of HD, months	28 (12–48)	15 (8–35)	31 (20–58)	0.01
Frequency of HD, no. of sessions per week				0.35
Two	4 (5.7)	2 (5.7)	2 (5.7)	
Three	53 (76)	29 (83)	24 (69)	
Four	10 (14)	4 (11)	6 (17)	
Five	3 (4.3)	0 (0)	3 (8.6)	
Vascular dialysis access				0.12
Arteriovenous fistula/graft	49 (70)	21 (60)	28 (80)	
Tunneled central venous catheter	21 (30)	14 (40)	7 (20)	
Previous kidney transplantation	5 (7.1)	2 (5.7)	3 (8.6)	1
Previous peritoneal dialysis	19 (27)	7 (20)	12 (34)	0.28
Comorbidities				
Ischaemic heart disease	11 (16)	7 (20)	4 (11)	0.51
Heart failure	12 (17)	7 (20)	5 (14)	0.75
Previous stroke	19 (27)	10 (29)	9 (26)	1
Peripheral vascular disease	22 (31)	16 (46)	6 (17)	0.02
Paroxysmal atrial fibrillation	19 (27)	8 (23)	11 (31)	0.59
Current medication				
ACEi/ARB	21 (30)	15 (43)	6 (17)	0.04
Loop diuretic	37 (53)	21 (60)	16 (46)	0.34
Beta-blocker	41 (59)	21 (60)	20 (57)	1
Selective calcium blocker	27 (39)	14 (40)	13 (37)	1
Digoxin	2 (2.9)	2 (5.7)	0 (0)	0.49
Lipid-lowering agent	29 (41)	19 (54)	10 (29)	0.05
Antiplatelet agent	31 (44)	19 (54)	12 (34)	0.15
Anticoagulant	10 (14)	5 (14)	5 (14)	1
BMI, kg/m^2^	27 (24–32)	27 (24–33)	27 (23–30)	0.31
Pre-dialysis BP				
Systolic, mmHg	142 (123–161)	141 (121–158)	144 (125–162)	0.97
Diastolic, mmHg	71 (64–81)	72 (64–81)	71 (64–86)	0.96

Data are presented as *n* (%) or median (IQR)

ACEi, ACE inhibitor; ARB, angiotensin II receptor blocker; HD, haemodialysis

**Table 2:** Baseline characteristics of participants with diabetes

Characteristic	Diabetes (*n*=35)
Type 1 diabetes	7 (20)
Duration of diabetes, years	20 (10–30)
HbA_1c_, mmol/mol	56±15
HbA_1c_, %	7.2±1.4
Home glucose monitoring	
BGM <1 per day	17 (49)
BGM ≥1 per day	9 (26)
Intermittently scanned CGM (Freestyle Libre^a^)	5 (14)
Real-time CGM (Dexcom G6^b^)	4 (11)
Hypoglycaemia awareness	
Reduced awareness	8 (23)
Uncertain	8 (23)
Aware	19 (54)
Insulin treatment	30 (86)
Short- or rapid-acting insulin	13 (37)
Long- or intermediate-acting insulin	29 (83)
Non-insulin glucose-lowering medication	14 (40)

Data are presented as *n* (%), mean ± SD or median (IQR)

^a^Abbott Diabetes Care, Alameda, USA

^b^Dexcom, San Diego, USA

BGM, blood glucose monitoring

**Table 3:** Baseline pre-dialysis blood sample and dialysis measures

Measurement	All (*n*=70)	Diabetes (*n*=35)	No diabetes (*n*=35)	*p* value
Pre-dialysis blood samples				
Haemoglobin, mmol/l	7.1±0.7	7.2±0.7	7.0±0.7	0.39
Leucocytes, 1×10^9^/l	7.3±2.4	7.8±2.6	6.9±2.1	0.10
Albumin, g/l	32±4.3	32±4.1	33±4.5	0.17
Potassium, mmol/l	4.5±0.6	4.3±0.5	4.7±0.5	0.01
Sodium, mmol/l	138±2.7	138±3.2	138±2.2	0.67
Ionised calcium, mmol/l	1.18±0.08	1.18±0.09	1.17±0.08	0.60
Phosphorous, mmol/l	1.65±0.45	1.65±0.44	1.64±0.46	0.91
Parathyroid hormone, pmol/l	28.0±21.2	25.7±19.2	30.3±23.1	0.37
Total CO_2_, mmol/l	24.0±3.0	24.6±3.3	23.5±2.6	0.14
C-reactive protein^a^, mg/l	5.0 (1.0–12.0)	7.1 (1.2–13.0)	2.9 (1.0–8.3)	0.08
Dialysis parameters				
Weekly Kt/V^a^	3.7±0.6	3.5±0.5	3.8±0.5	0.07
Dialysate potassium, mmol/l	2 (2–2)	2 (2–2)	2 (2–2)	0.33
Dialysate calcium, mmol/l	1.25 (1.25–1.25)	1.25 (1.25–1.25)	1.25 (1.25–1.25)	0.65
Dialysate sodium, mmol/l	138 (138–138)	138 (138–138)	138 (138–138)	0.39
Dialysate bicarbonate, mmol/l	36 (34–38)	36 (34–38)	38 (34–38)	0.45
Dialysate temperature, °C	36.5 (36.5–37.0)	36.5 (36.5–37.0)	36.5 (36.5–37.0)	0.68
Dry weight target, kg	85±20	85±18	84±22	0.84
Ultrafiltration rate, ml kg^−1^ h^−1^	4.5 (0.7–8.2)	2.9 (1.2–7.9)	5.1 (0.9–8.2)	0.38

Data are presented as mean ± SD or median (IQR)

^a^Missing observations: C-reactive protein (*n*=5); and weekly Kt/V (*n*=13). Kt/V was calculated as (K × t) / V, where K is the dialyser clearance of urea (ml/min), t is the dialysis duration (min) and V is the volume of distribution of urea

### Cardiac arrhythmias

During follow-up, 44 (63%) participants had at least one event of any arrhythmia and 23 (33%) had at least one clinically significant arrhythmia, with the same proportion among participants with and without diabetes (Table 4). A total of 1347 clinically significant arrhythmic events were detected. Most of these were bradyarrhythmias, of which one-third (469 events) were pauses. Nearly 90% of the bradyarrhythmia events were detected in participants without diabetes and were largely driven by a high number of events in a few participants. The distribution of arrhythmic events per participant is shown in electronic supplementary material (ESM) Fig. 1. Of the 12 observed ventricular tachycardia events, three were sustained. No episodes of ventricular fibrillation were observed.

**Table 4:** Cardiac arrhythmias

Arrythmia	All (*n*=70)	Diabetes (*n*=35)	No diabetes (*n*=35)
Any arrhythmia			
Participants with at least one event	44 (63)	22 (63)	22 (63)
Clinically significant arrhythmias			
Participants with at least one event	23 (33)	12 (34)	11 (31)
Clinically significant arrhythmia subtype			
Bradyarrhythmia			
Participants with at least one event	18 (26)	8 (23)	10 (29)
No. of events	1335	135	1200
Events per participant with bradyarrhythmia	9 (6–51)	8 (7–17)	30 (3–103)
Non-sustained ventricular tachycardia			
Participants with at least one event	5 (7)	4 (11)	1 (3)
No. of events	9	8	1
Events per participant with non-sustained ventricular tachycardia	2 (1–2)	2 (2–2)	1 (1–1)
Sustained ventricular tachycardia			
Participants with at least one event	2 (3)	2 (6)	0
No. of events	3	3	0
Events per participant with sustained ventricular tachycardia	2 (1–2)	2 (1–2)	0
Ventricular fibrillation			
No. of events	0	0	0
Atrial fibrillation			
Participants with at least one event	27 (39)	13 (37)	14 (40)
No. of events	1177	731	446
Events per participant with atrial fibrillation	5 (1–44)	3 (1–39)	7 (3–46)
Other supraventricular tachycardia			
Participants with at least one event	18 (26)	7 (20)	11 (31)
No. of events	348	24	324
Events per participant with supraventricular tachycardia	2 (1–6)	2 (1–3)	2 (2–8)

Data are presented as *n* (%) or median (IQR)

Atrial fibrillation and other supraventricular tachycardia were detected in 27 (39%) and 18 (26%) participants, respectively. Of the 51 participants without a history of paroxysmal atrial fibrillation, 15 (29%) were diagnosed with new-onset atrial fibrillation during the study, including nine (38%) participants with diabetes and six (22%) participants without diabetes. Based on our findings, four (6%) participants were referred for pacemaker implantation during follow-up and nine (13%) participants had their anti-arrhythmia treatment adjusted. This included initiation and dose adjustments of beta-blockers and digoxin due to atrial fibrillation and other supraventricular arrhythmias, and discontinuation of beta-blockers due to bradyarrhythmias.

### Continuous glucose monitoring

A total of 4414 days of CGM data were obtained during follow-up, including 2045 days for the diabetes group and 2369 days for the group without diabetes. The median time that the CGM device was worn per period was 9 days (IQR 8–10 days). The median percentage of time spent at 3.9–10.0 mmol/l was 45% (IQR 32–64%) for the diabetes group and 96% (IQR 94–98%) for the group without diabetes (Table 5). The median time spent in hyperglycaemia (>10.0 mmol/l) was 54% (IQR 35–68%) and 2.4% (IQR 1–6%) for participants with and without diabetes, respectively, and the median time in hypoglycaemia (<3.9 mmol/l) was 0.4% (IQR 0.0–1.0%) and 0.6% (IQR 0.0–1.2%).

**Table 5:** Glycaemic characteristics based on CGM

Characteristic	Diabetes (*n*=35)	Non-diabetes (*n*=35)
Data obtainment		
No. of CGM periods per participant	8 (6–9)	9 (8–9)
Days CGM are worn per period	9 (8–10)	9 (8–10)
Time CGM active, %	98 (93–99)	98 (95–100)
Time in range, %		
Time above range >13.9 mmol/l	17.3 (5.7–31.7)	0.0 (0.0–0.1)
Time above range >10.0 mmol/l	54.1 (35.1–68.1)	2.4 (0.9–5.8)
Time in range 3.9–10.0 mmol/l	44.6 (31.7–64.3)	96.4 (94.0–97.6)
Time below range <3.9 mmol/l	0.4 (0.0–1.0)	0.6 (0.0–1.2)
Time below range <3.0 mmol/l	0.0 (0.0–0.3)	0.0 (0.0–0.2)
Mean sensor glucose, mmol/l	11.0±2.6	6.7±0.6
Glycaemic variability		
SD, mmol/l	3.1±1.0	1.4±0.4
CV, %	28.7±6.8	20.9±4.0
Hypoglycaemic events		
Participants with at least one event (level 1 or 2)	27 (77)	32 (91)
Participants with at least one level 2 event	23 (65)	25 (71)
No. of events (level 1 or 2)	325	539
No. of level 2 events	121	149
Events per participant with hypoglycaemic events	8 (3–15)	12 (4–24)
Duration per hypoglycaemic event, min	40 (20–70)	25 (15–45)
Mean sensor glucose during hypoglycaemic events, mmol/l	3.3 (3.0–3.6)	3.3 (2.9–3.6)

Data are presented as *n* (%), mean ± SD or median (IQR)

There were 864 hypoglycaemic events, and most occurred among participants without diabetes. Nearly one-third of the hypoglycaemic events were level 2 events. The median duration of hypoglycaemic events was longer for the participants with diabetes than for those without diabetes, while the mean sensor glucose during events was similar for the two groups. The distribution of hypoglycaemic events per participant is shown in ESM Fig. 2, while ESM Table 1 provides CGM data stratified by type 1 and type 2 diabetes.

### Association between cardiac arrhythmias and glycaemic measures

Out of a total of 234 arrhythmic events with concomitantly available CGM data, there were nine individual observation hours with concurrent arrhythmic event and hypoglycaemic event in four individuals. This included one event in a participant with diabetes and eight events in three participants without diabetes. For the participant with diabetes, the one hypoglycaemic event with concurrent arrhythmia was a level 1 event. For the participants without diabetes, three of the eight hypoglycaemic events with concurrent arrhythmia were level 2 events.

**Fig. 2:**
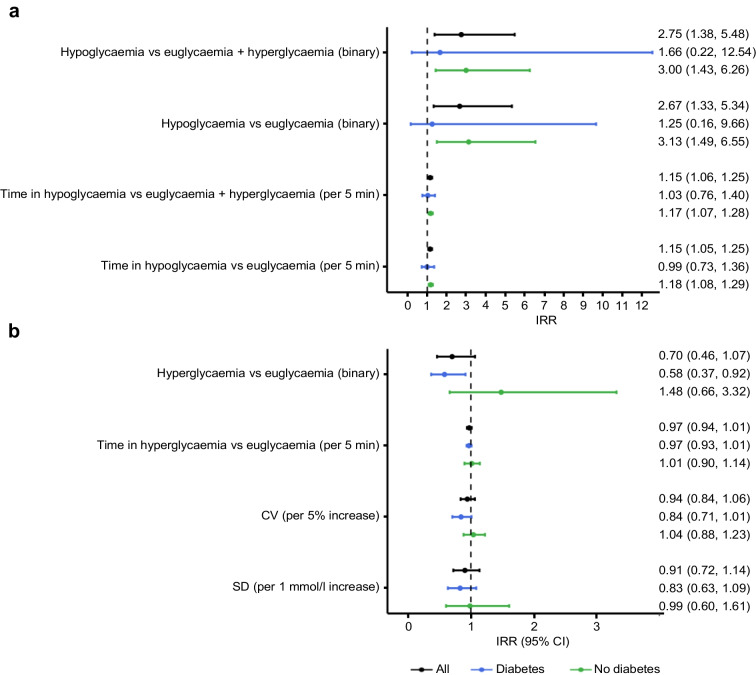
IRR (95% CI) of arrhythmias in relation to within-subject changes in glycaemic measures regarding hypoglycaemia (**a**) and hyperglycaemia and glycaemic variability (**b**)

Figure 2a shows the IRRs with 95% CI of arrhythmias in relation to hypoglycaemia. An hour with the occurrence of hypoglycaemia was associated with an increased rate of arrhythmias compared with an hour of euglycaemia in the participants without diabetes (IRR 3.13 [95% CI 1.49, 6.55]), but not in those with diabetes (IRR 1.25 [95% CI 0.16, 9.66]). Similarly, increasing time spent in hypoglycaemia within the same hour was associated with an increased rate of arrhythmias compared with euglycaemia among the participants without diabetes (IRR 1.18 [95% CI 1.08, 1.29] per 5 min), but not among those with diabetes (IRR 0.99 [95% CI 0.73, 1.36] per 5 min). These findings were confirmed when comparing hypoglycaemia with euglycaemia and hyperglycaemia combined.

The IRRs of arrhythmias in relation to hyperglycaemia and glycaemic variability are shown in Fig. 2b. An hour with the occurrence of hyperglycaemia was associated with a decreased rate of arrhythmias compared with an hour of euglycaemia in the participants with diabetes (IRR 0.58 [95% CI 0.37, 0.92]), whereas there was no clear association between arrhythmias and hyperglycaemia in those without diabetes (IRR 1.48 [95% CI 0.66, 3.32]). No association was found between arrhythmias and time spent in hyperglycaemia within same hour for either of the groups. Glycaemic variability measured as CV and SD within the same day showed no association with the rate of arrhythmias in participants with or without diabetes. Furthermore, we found no association between the Δ plasma glucose and arrhythmias for either of the groups (ESM Fig. 3). The analyses stratified by daytime and night-time are presented in ESM Fig. 4. Overall, the effect of the different glycaemic measures on the rate of arrhythmias was similar for daytime and night-time.

### Additional analyses

We found no association between arrhythmias and the baseline use of beta-blockers in the total sample (IRR 0.86 [95% CI 0.30, 2.47]), nor in those with diabetes (IRR 0.31 [95% CI 0.07, 1.30]) or those without diabetes separately (IRR 2.25 [95% CI 0.51, 9.81]). Furthermore, there was no association between arrhythmias and the baseline use of selective calcium blockers in the total sample (IRR 1.03 [95% 0.35, 3.00]), nor in those with diabetes (IRR 1.22 [95% CI 0.28, 5.42]) or those without diabetes separately (IRR 1.14 [95% CI 0.24, 5.55]).

## Discussion

This study provides extensive data on simultaneous long-term continuous arrhythmic and glycaemic monitoring from individuals with and without diabetes receiving haemodialysis. Clinically significant arrhythmias were detected in 34% of the participants with diabetes and 31% of those without diabetes. Atrial fibrillation episodes were detected in nearly 40% of the participants, and 21% were diagnosed with new-onset atrial fibrillation. Hypoglycaemia was observed in 77% of those with diabetes and 91% of those without diabetes. Hypoglycaemia was associated with an increased rate of arrhythmias in participants without diabetes, whereas hyperglycaemia was associated with a decreased rate of arrhythmias in participants with diabetes. Glycaemic variability showed no association with arrhythmias regardless of the presence of diabetes.

Clinically significant arrhythmias were common and there were similar incidences of at least one event occurring among those with and without diabetes. Notably, bradyarrhythmia accounted for a much higher proportion of the clinically significant arrhythmias than ventricular tachycardia. Atrial fibrillation and other supraventricular tachycardias were also frequently observed. Three previous studies employing ILRs among individuals receiving haemodialysis used similar definitions of arrhythmias to those used in the present study [22–24]. The proportion of participants with at least one arrhythmic event in these studies ranged from 14% to 20% for non-sustained ventricular tachycardia, from 20% to 30% for bradyarrhythmia and from 28% to 41% for atrial fibrillation. These findings are similar to our own regarding the incidence of bradyarrhythmia and atrial fibrillation, although we found a lower incidence of non-sustained ventricular tachycardia. Studies in individuals with cardiovascular risk factors but not on haemodialysis found a similar incidence of new-onset atrial fibrillation but a lower incidence of bradyarrhythmia compared with our population [25, 26].

Interestingly, CGM-measured hypoglycaemic events were more frequent among the participants without diabetes than those with diabetes. This was true both in terms of the proportion of participants with at least one event and the number of events detected during follow-up. Although it should be noted that we had more days of CGM data from the participants without diabetes, these participants also spent a higher percentage of time in hypoglycaemia than those with diabetes, which accounts for the difference in days of CGM data.

In general, individuals with advanced CKD have a high risk of hypoglycaemia due to decreased renal gluconeogenesis, impaired insulin clearance and degradation, impaired counterregulatory hormone responses, and nutritional deprivation [17, 18]. To avoid hypoglycaemia, international consensus guidelines recommend that individuals with diabetes and CKD be less tightly regulated [27]. Specifically, it is recommended that they spend >50% of the time at 3.9–10.0 mmol/l and <1% below 3.9 mmol/l. Our diabetes population spent most time in hyperglycaemia (>10 mmol/l), which may at least partly explain why they had fewer hypoglycaemic events than the group without diabetes. A previous study applying CGM in individuals with CKD similarly observed a higher occurrence of hypoglycaemia in participants without diabetes than in those with diabetes [28]. Furthermore, in a recent study of 18 individuals on haemodialysis without diabetes, the median percentage of time spent below 3.9 mmol/l was 1.9% [29], which underscores the notion that hypoglycaemia is common in this population.

Hypoglycaemia has previously been linked to an increased risk of cardiovascular morbidity and mortality in individuals with CKD and in an incident haemodialysis population with and without diabetes [30, 31]. Our findings add to existing knowledge by showing a temporal relationship between hypoglycaemia and arrhythmias in individuals without diabetes receiving haemodialysis, but not in individuals with diabetes. The discrepancy in results between individuals with and without diabetes may be caused by differences in hypoglycaemia-related counterregulatory responses. It has previously been shown that a long duration of diabetes is associated with a lower hormonal response to hypoglycaemia and smaller ECG changes [32, 33]. Thus, hypoglycaemia in individuals without diabetes may elicit a greater counterregulatory response associated with higher cardiovascular risk.

For the participants with diabetes, hyperglycaemia was associated with a decreased rate of arrhythmias. This contrasts with the findings of three previous studies in individuals with diabetes but not on chronic maintenance haemodialysis. Specifically, a prior study in individuals with type 1 diabetes found that hyperglycaemia was associated with an increased rate of arrhythmias during the daytime [13], while two other studies in type 1 and type 2 diabetes found no association between hyperglycaemia and arrhythmias [12, 34]. Apart from the presence of severe CKD with the need for haemodialysis in our study, this discrepancy may be explained by differences in glycaemic management between the participants in the different studies. While our study participants with diabetes spent most time in hyperglycaemia, the study participants in the three aforementioned studies spent most time in euglycaemia. Data from two other studies in critically ill individuals suggest that the effect of acute hyperglycaemia on mortality is dependent on preexisting glycaemic management and that correction of chronic hyperglycaemia may be harmful in individuals with preexisting diabetes and a high HbA_1c_ [35, 36].

A phenomenon that potentially accounts for these observations is ‘relative hypoglycaemia’, where the glycaemic threshold for detecting and reacting to hypoglycaemia is elevated in individuals with chronic hyperglycaemia. The underlying mechanisms for this phenomenon remain uncertain but it has been suggested that sustained exposure to hyperglycaemia impairs glucose sensing by glucose-responsive neurons [37]. Consequently, these neurons perceive hypoglycaemia at elevated glucose levels, thereby causing hypoglycaemia-related counterregulatory responses to be triggered at normal blood glucose concentrations in individuals with relative hypoglycaemia. Thus, in our diabetes cohort, the glycaemic threshold for hypoglycaemia perception may be raised and a falling blood glucose level from hyperglycaemia to euglycaemia might trigger the counterregulatory responses associated with arrhythmias. This also serves as a possible explanation for the lack of association between arrhythmias and hypoglycaemia in these participants.

Our results are an important reminder that hypoglycaemia is common in individuals receiving haemodialysis, particularly those without diabetes, and may cause serious complications. The proportion of individuals with hypoglycaemia in our study is higher than previously reported in a haemodialysis population [31], underscoring the importance of applying CGM when evaluating hypoglycaemia. However, CGM-measured hypoglycaemia is also common in the general population without diabetes. A study of 153 healthy individuals without diabetes found that they spent 1.1% of the time below 3.9 mmol/l [38]. Our haemodialysis population did not spend more time in hypoglycaemia than this general population without diabetes but individuals receiving haemodialysis might be particularly vulnerable to hypoglycaemia due to underlying predisposing factors for arrhythmias. In addition, while major clinical trials have demonstrated that severe hypoglycaemia (requiring assistance from another person) is associated with adverse cardiovascular events, our data suggest that in the haemodialysis population, non-severe hypoglycaemic events should also be considered as potentially harmful. This is consistent with data from a recent study that reported an association between hypoglycaemic events at any level of severity and cardiovascular outcomes [39].

Based on our findings, we recommend considering CGM and long-term ECG monitoring for individuals receiving chronic maintenance haemodialysis with unexplained symptoms that could be caused by hypoglycaemia or arrhythmia (e.g. fatigue, dizziness, palpitations or dyspnoea). Especially for those without diabetes, hypoglycaemia might be overlooked. However, it remains unclear whether hypoglycaemia-related complications are causative or whether hypoglycaemia is merely a marker of vulnerability. Further studies are needed to determine whether prevention of hypoglycaemia can prevent arrhythmic events in individuals on haemodialysis.

The strengths of this study include the combination of long-term continuous heart rhythm monitoring with CGM followed by assessment of the temporal association between arrhythmias and glycaemic measures, where each participant served as their own control in the analyses. The random effect for participant ID ensured that the results were not disproportionally influenced by participants with a high number of events. Individuals with paroxysmal atrial fibrillation were included because they represent approximately one-third of the haemodialysis population regardless of diabetes status [22–24, 40]. The sex distribution of the total sample was similar to previous reports [40]; however, there was a higher proportion of women among those with diabetes and a higher proportion of men among those without diabetes. Although not directly impacting the analyses concerning the association between arrhythmias and glycaemic measures, the sex distribution of our study population may limit the generalisability of our findings. Thus, we believe the study population is representative of the general haemodialysis population with a similar distribution of sex and ethnicity.

One of the limitations is that the observational study design precludes causal inference. CGM data were only collected for a part of the follow-up period due to the practical burden on the participants related to CGM use, and there were relatively few arrhythmic events during CGM. Furthermore, our sample size was relatively small. This led to wide CIs for the IRRs, and the study cannot exclude an association between arrhythmias and any of the glycaemic measures. Accordingly, we did not stratify the analyses by arrhythmic subtype, levels of hypoglycaemia, or sex.

For some participants, the anti-arrhythmic medication was changed during follow-up; this may have impacted the incidence of arrhythmias. Furthermore, a part of the diabetes population had their own personal non-blinded CGM device, which may have reduced the occurrence of hypoglycaemia for these participants. The dialysate glucose concentration was standardised at 5.6 mmol/l for all participants but dialysate electrolyte concentrations could be altered during the study, including during CGM periods. These circumstances reflect the real-life setting and are unlikely to have impacted the results concerning the association between arrhythmias and glycaemic measures. The performance of the Dexcom G6 Pro CGM system has been found to be reasonably accurate in a haemodialysis population with diabetes [41].

### Conclusion

This long-term observational study of individuals receiving haemodialysis found that arrhythmias and hypoglycaemia were common in both those with and without diabetes. We found an increased rate of arrhythmias during hypoglycaemia in individuals without diabetes and a decreased rate of arrhythmias during hyperglycaemia in individuals with diabetes, which suggests a temporal relationship between arrhythmias and glucose level in those undergoing haemodialysis regardless of diabetes status. Increased awareness of arrhythmias and hypoglycaemia in both individuals with and without diabetes may improve the prognosis of this high-risk population.

## Supplementary Information

Below is the link to the electronic supplementary material.

Supplementary file1 (PDF 383 KB)

## Data Availability

The data of this study are not publicly available due to the risk of potential re-identification of individuals but are available from the corresponding author upon reasonable request.

## Abbreviations

CGM: Continuous glucose monitoring
CKD: Chronic kidney disease
ILR: Implantable loop recorder
IRR: Incidence rate ratio
