# Supplementary material for: Cardiac arrhythmia and hypoglycaemia among individuals with and without diabetes receiving haemodialysis (the CADDY study): a Danish multicentre cohort study
Source: Diabetologia. 2025 Feb 28;68(6):1126–39. doi: 10.1007/s00125-025-06388-5 (PMC12069408; doi:10.1007/s00125-025-06388-5)

Electronic supplementary material (ESM)

ESM Table 1. Glycaemic characteristics based on continuous glucose monitoring stratified by type 1 and type 2 diabetes.

ESM Figure 1. Number of clinically significant arrhythmia events (Panel A), atrial fibrillation events (Panel B), and other supraventricular tachycardia events (Panel C) for participants with at least one event.

ESM Figure 2. Number of hypoglycaemic events for participants with at least one event.

ESM Figure 3. Incidence rate ratio (IRR) (95% CI) of arrhythmias in relation to within-subject changes in glycaemic measures regarding delta plasma glucose (two-hour absolute excursion in plasma glucose).

ESM Figure 4. Incidence rate ratio (IRR) (95% CI) of arrhythmias in relation to within-subject changes in glycaemic measures regarding hypoglycaemia, hyperglycaemia, and glycaemic variability stratified by daytime and nighttime.

ESM Table 1. Glycaemic characteristics based on continuous glucose monitoring stratified by type 1 and type 2 diabetes.

	**Type 1 diabetes (n=7)**	**Type 2 diabetes (n=28)**
Time in ranges (%)		
Time above range >13.9 mmol/L	32.0 (26.9–37.0)	15.0 (4.7–22.9)
Time above range >10.0 mmol/L	65.6 (60.2–69.1)	50.1 (30.1–64.8)
Time in range 3.9–10.0 mmol/L	33.4 (29.8–39.3)	49.5 (35.3–69.3)
Time below range <3.9 mmol/L	1.0 (0.4–1.5)	0.2 (0.0–0.7)
Time below range <3.0 mmol/L	0.0 (0.0–0.2)	0.0 (0.0–0.3)
Mean sensor glucose (mmol/l)	11.9±1.5	10.8±2.7
Glycaemic variability		
Standard deviation (mmol/l)	4.3±1.0	2.9±0.8
Coefficient of variation (%)	35.4±4.1	27.0±6.2
Hypoglycaemic events		
Patients with at least one event (level 1 or 2)	7 (100)	20 (71)
Number of events (level 1 or 2)	96	229
Events per patient with hypoglycaemic events	10 (8–19)	8 (2–13)
Duration per hypoglycaemic event (min)	35 (20–60)	45 (20–75)
Mean sensor glucose during hypoglycaemic events (mmol/L)	3.4 (3.1–3.6)	3.3 (2.9–3.5)

Data are presented as n (%), mean ± SD or median (IQR).

ESM Figure 1. Number of clinically significant arrhythmia events (Panel A), atrial fibrillation events (Panel B), and other supraventricular tachycardia events (Panel C) for participants with at least one event.

A. Clinically significant arrhythmias

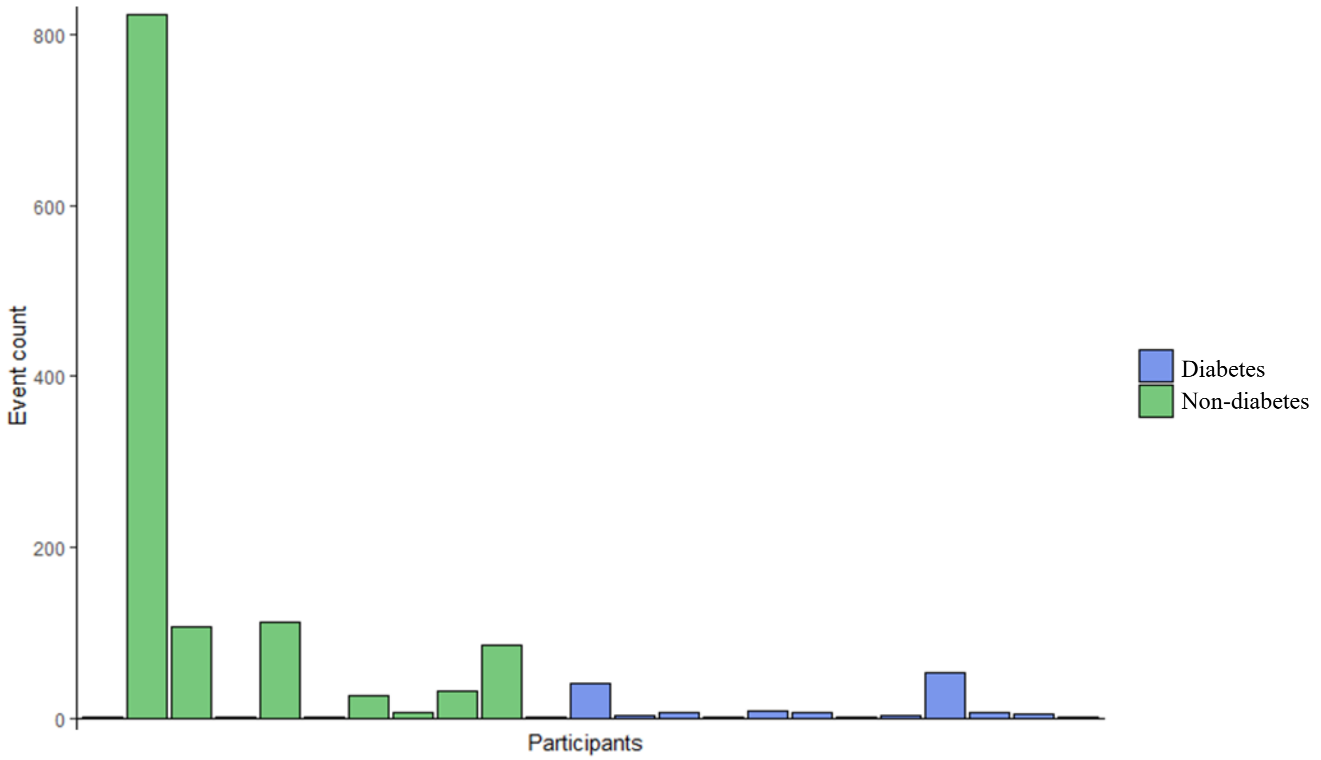

B. Atrial fibrillation

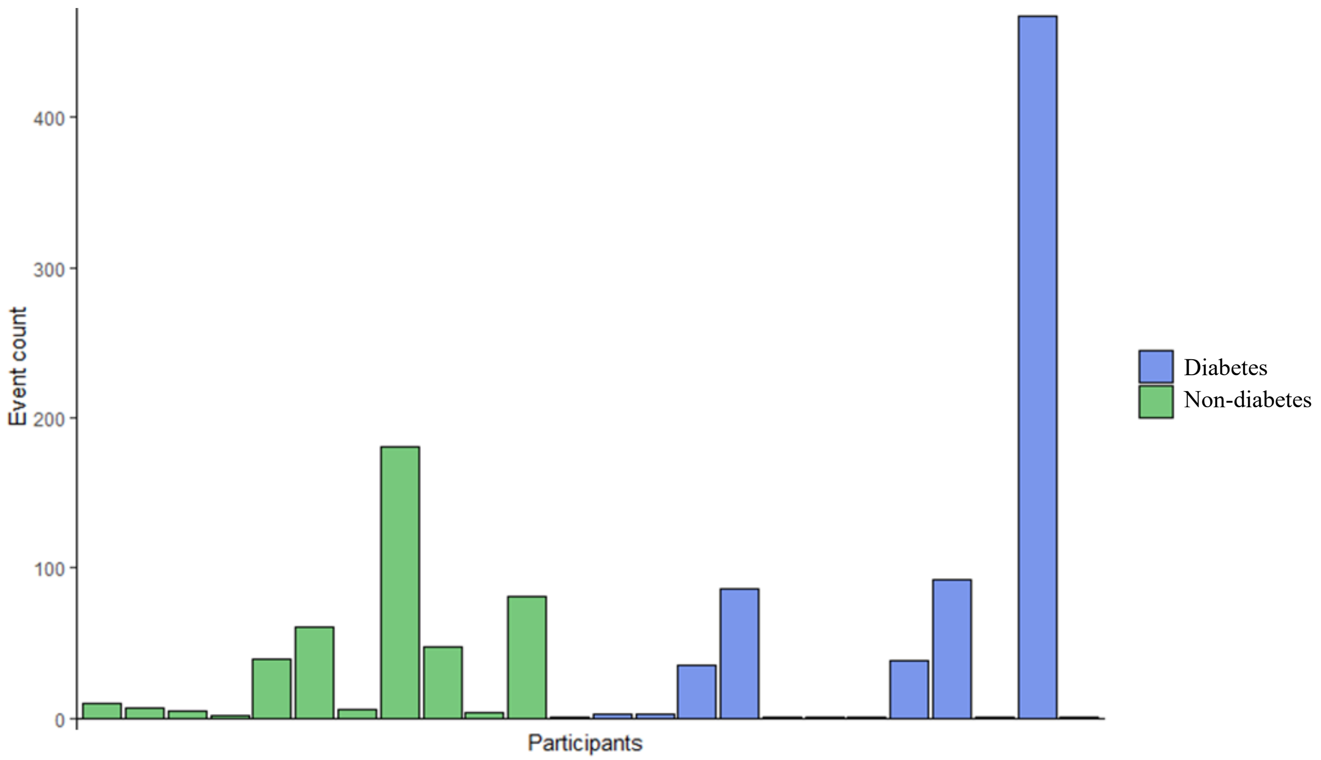

C. Other supraventricular tachycardia

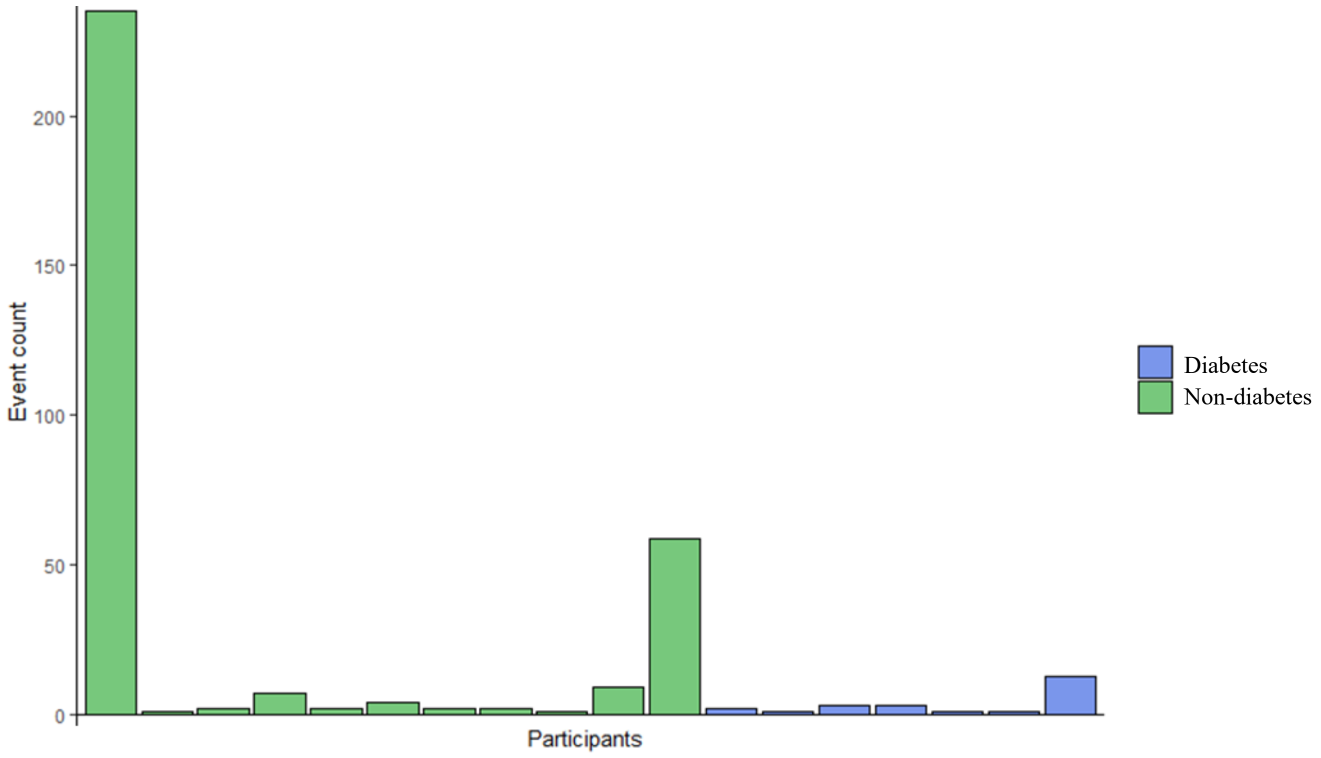

ESM Figure 2. Number of hypoglycaemic events for participants with at least one event.

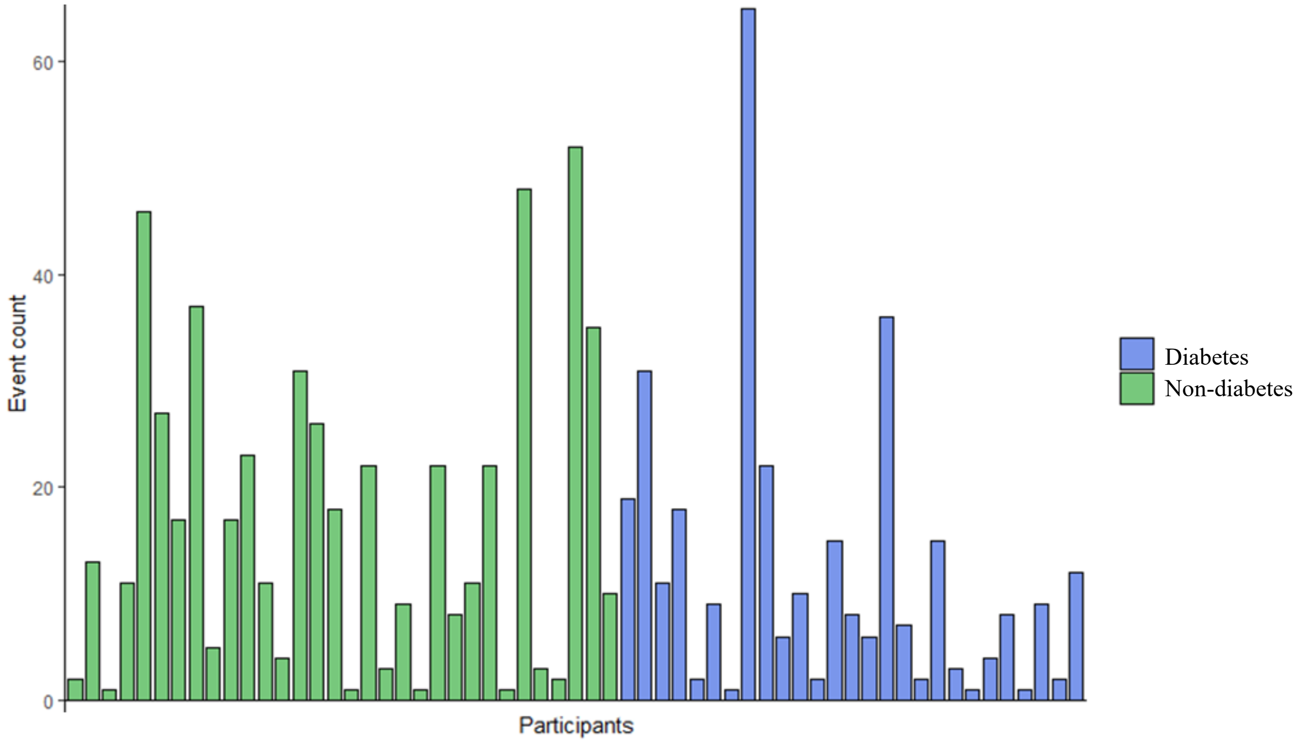

ESM Figure 3. Incidence rate ratio (IRR) (95% CI) of arrhythmias in relation to within-subject changes in glycaemic measures regarding delta plasma glucose (two-hour absolute excursion in plasma glucose).

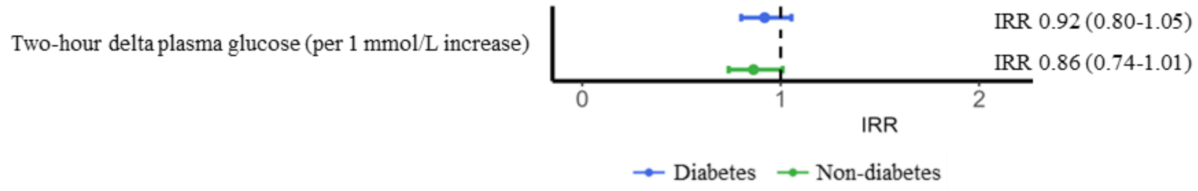

ESM Figure 4. Incidence rate ratio (IRR) (95% CI) of arrhythmias in relation to within-subject changes in glycaemic measures regarding hypoglycaemia, hyperglycaemia, and glycaemic variability stratified by daytime and nighttime.

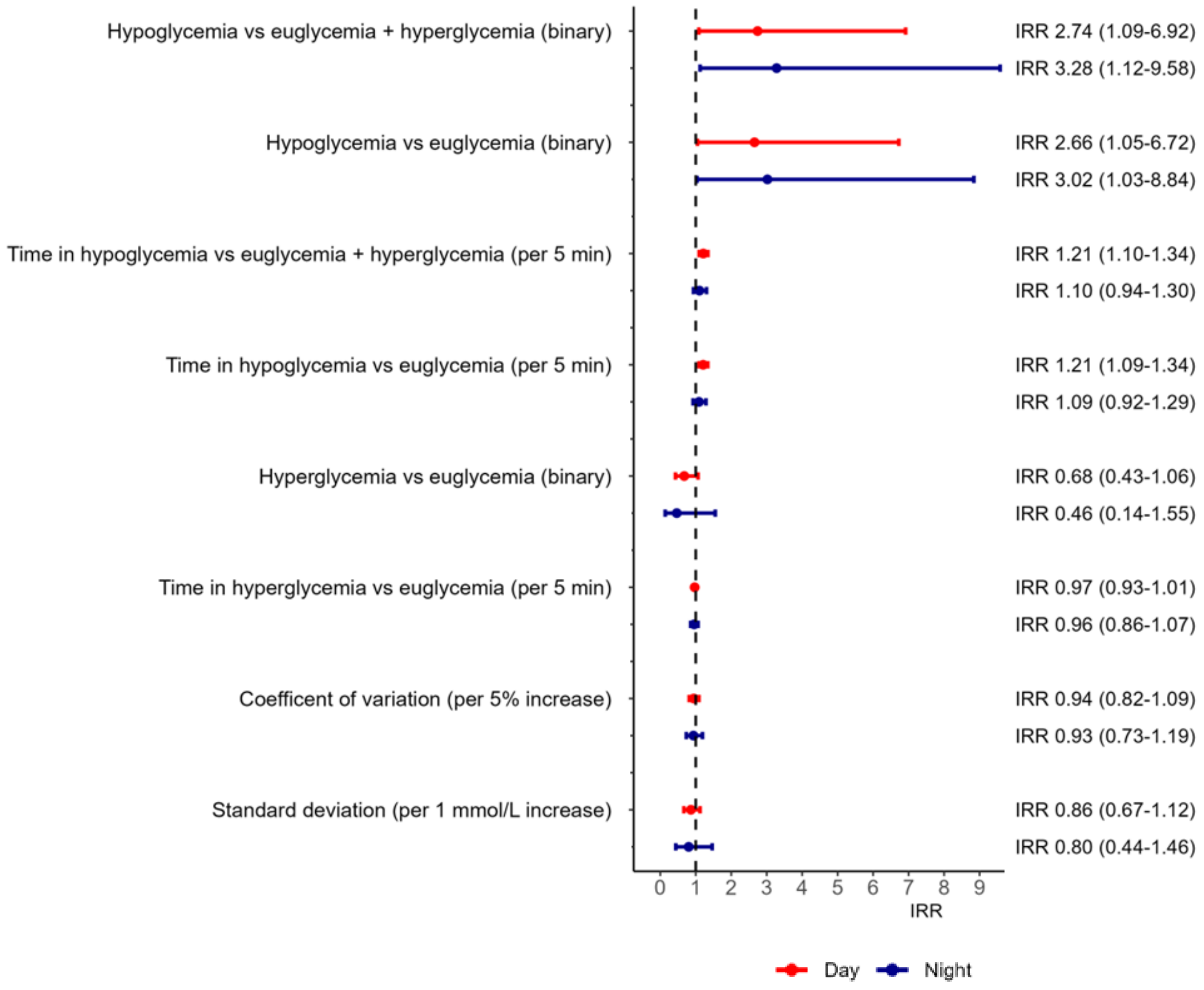

Supplement: Supplementary file 1 — Supplementary file1 (PDF 383 KB) [file 125_2025_6388_MOESM1_ESM.pdf]
